# HIV-1 non-macrophage-tropic R5 envelope glycoproteins are not more tropic for entry into primary CD4+ T-cells than envelopes highly adapted for macrophages

**DOI:** 10.1186/s12977-015-0141-0

**Published:** 2015-03-14

**Authors:** Thomas Musich, Olivia O’Connell, Maria Paz Gonzalez-Perez, Cynthia A Derdeyn, Paul J Peters, Paul R Clapham

**Affiliations:** Program in Molecular Medicine, University of Massachusetts Medical School, 373, Plantation Street, Worcester, MA 01605 USA; Department of Pathology and Laboratory Medicine, Emory Vaccine Center at Yerkes National Primate Center, Emory University, 954 Gatewood Road, Atlanta, GA 30329 USA; Department of Microbiology and Physiological Systems, University of Massachusetts Medical School, 55 N. Lake Ave, Worcester, MA 01655 USA

**Keywords:** HIV-1, Tropism, Macrophage-tropism, Non-macrophage-tropism, Dendritic cells, Trans-infection, Envelope, T-cells, Macrophages, Functionality

## Abstract

**Background:**

Non-mac-tropic HIV-1 R5 viruses are predominantly transmitted and persist in immune tissue even in AIDS patients who carry highly mac-tropic variants in the brain. Non-mac-tropic R5 envelopes (Envs) require high CD4 levels for infection contrasting with highly mac-tropic Envs, which interact more efficiently with CD4 and mediate infection of macrophages that express low CD4. Non-mac-tropic R5 Envs predominantly target T-cells during transmission and in immune tissue where they must outcompete mac-tropic variants. Here, we investigated whether Env+ pseudoviruses bearing transmitted/founder (T/F), early and late disease non-mac-tropic R5 envelopes mediated more efficient infection of CD4+ T-cells compared to those with highly mac-tropic Envs.

**Results:**

Highly mac-tropic Envs mediated highest infectivity for primary T-cells, Jurkat/CCR5 cells, myeloid dendritic cells, macrophages, and HeLa TZM-bl cells, although this was most dramatic on macrophages. Infection of primary T-cells mediated by all Envs was low. However, infection of T-cells was greatly enhanced by increasing virus attachment with DEAE dextran and spinoculation, which enhanced the three Env+ virus groups to similar extents. Dendritic cell capture of viruses and trans-infection also greatly enhanced infection of primary T-cells. In trans-infection assays, non-mac-tropic R5 Envs were preferentially enhanced and those from late disease mediated levels of T-cell infection that were equivalent to those mediated by mac-tropic Envs.

**Conclusions:**

Our results demonstrate that T/F, early or late disease non-mac-tropic R5 Envs do not preferentially mediate infection of primary CD4+ T-cells compared to highly mac-tropic Envs from brain tissue. We conclude that non-macrophage-tropism of HIV-1 R5 Envs in vitro is determined predominantly by a reduced capacity to target myeloid cells via low CD4 rather than a specific adaptation for T-cells entry that precludes macrophage infection.

**Electronic supplementary material:**

The online version of this article (doi:10.1186/s12977-015-0141-0) contains supplementary material, which is available to authorized users.

## Background

HIV-1 infects cells expressing CD4 and either CCR5 or CXCR4 [1]. HIV-1 isolates that use CCR5 were believed to infect both T-cells and macrophages and were classified as macrophage-tropic (M-tropic), while isolates using CXCR4 preferentially infected T-cells and were described as T-tropic [1]. However, the term ‘M-tropic’ to describe all CCR5-using variants is no longer valid since it has become clear that they vary extensively in their ability to infect macrophages [2-10]. Macrophage-tropic (mac-tropic) R5 strains can still replicate despite low levels of cell surface CD4 on macrophages for infection, while non-macrophage-tropic (non-mac-tropic) R5 viruses require high amounts of CD4, presumably limiting them to CD4+ T-cells that express significantly more CD4 compared to macrophages [2,3,11]. The majority of transmitted or founder viruses described are non-mac-tropic R5 viruses [12-17] and viruses with this phenotype remain predominant in immune tissue [2,3] even at late stages of disease when mac-tropic variants can be increasingly detected in blood [18-20] and brain tissue [2,3,5,11,21-25]. These observations emphasize that CD4+ T-cells are the major cellular targets for HIV during transmission and throughout disease in immune tissue.

Determinants that modulate R5 macrophage infection have been mapped to residues within or proximal to the CD4 binding site on gp120 of the HIV-1 envelope glycoprotein (Env) [11,26,27] as well as in the variable V1 [28], V2 and [29] V3 loops [26] at the apex of the trimer [30]. These data are consistent with mac-tropic R5 Envs carrying a higher affinity for CD4, which includes increased initial binding of CD4 by the outer domain on gp120 but also more efficient triggering of the conformational changes that shift the V1V2 loops and open the trimer to enable CD4 to recruit determinants on the V1V2 stem to form the bridging sheet [31]. Our studies with neutralizing mabs that target the V2 and V3 loops support this interpretation and hint at structural changes in highly mac-tropic R5 Envs at the trimer apex that facilitate CD4 induced opening and formation of the bridging sheet and coreceptor binding site [31].

The presence of highly mac-tropic Envs in brain tissue represents an adaptation for replication in macrophages and microglia, which form the main HIV reservoir there. However, the factors in immune tissue that preferentially favor high CD4-requiring, non-macrophage-tropic R5 viruses rather than variants that interact efficiently with CD4 are less clear. We previously hypothesized [8,31] that non-macrophage-tropic Envs in immune tissue evolve Env trimers that are tightly closed with poor access to the CD4 binding site to protect against neutralizing antibodies. These Envs will require high concentrations of CD4 to trigger opening and assembly of the coreceptor binding site. In contrast, mac-tropic Envs in the immunoprivileged environment of the brain are only exposed to low antibody concentrations [32-34] and can evolve to open more readily in response to low CD4 levels. Surprisingly, we failed to show a significant difference in the sensitivity of non-mac-tropic Env+ pseudovirions to heterologous neutralizing antibodies in HIV-1+ human sera compared to those carrying highly mac-tropic brain-derived Envs [31]. In addition, the maintenance of the non-mac-tropic R5 Env phenotype during transmission and in the acute phase [12,14-17] when neutralizing antibodies (nabs) are usually absent, suggest the presence of additional selective forces that favor non-mac-tropic R5 viruses. An alternative hypothesis is that non-mac-tropic R5 Envs have evolved to mediate maximal replication in T-cells (rather than macrophages) i.e. they are T-cell tropic, even though they confer a lower Env:CD4 affinity compared to mac-tropic R5 Envs.

Here, we investigated whether non-mac-tropic R5 Envs mediate an enhanced tropism for T-cells. Our data show that non-mac-tropic R5 Envs either from early or late disease do not mediate more efficient infection of CD4+ T-cells compared to mac-tropic Envs, even though they are likely to predominantly target T-cells and dominate over more mac-tropic variants in immune tissue in vivo. These results point to additional unknown selective pressures that enable non-mac-tropic R5 viruses to out-compete mac-tropic variants in immune tissue throughout disease.

## Results

### Infection of macrophages and HeLa TZM-bl cells

**Figure 1:**
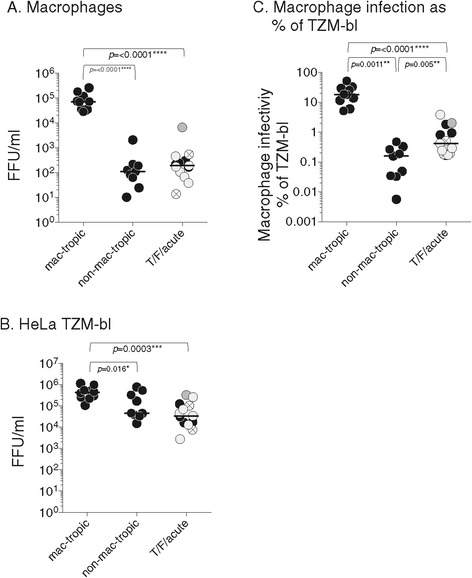
**Env+ pseudovirion infection of HeLa TZM-bl and macrophages. (A)** Macrophage-tropic R5 Envs mediate 100-1000-fold higher infection of macrophages compared to T/F/acute and late disease non-mac-tropic R5 Envs. **(B)** Several late disease non-mac-tropic Envs and most T/F/acute Envs mediated lower infection of HeLa TZM-bl cells compared to macrophage tropic Envs. **(C)** Plotting macrophage infectivity titers as a percent of TZM-bl titers suggests that T/F/acute Envs are not as non-mac-tropic as late disease non-mac-tropic Envs. Significant differences were evaluated using Mann Whitney or Wilcoxon matched pair tests as described in Materials and Methods. Pseudoviruses carrying clade B Envs are represented by black symbols, those with clade C Envs by light gray symbols, clade A by hatched symbols and CRF A/C by dark gray. Median values are marked.

We first compared Env+ GFP-reporter pseudovirion infection of primary macrophages with infectivity for TZM-bl cells (Figure 1). Mac-tropic Envs mediated high levels of infectivity for macrophages as expected with infectivity titers that were several orders of magnitude higher than for non-mac-tropic late disease Envs (Figure 1A). Of the T/F/acute R5 Envs, only R66M conferred a moderately high level of macrophage infection albeit still at a level lower than the highly mac-tropic R5 Envs.

HeLa TZM-bl cells express high levels of CD4 and CCR5 and are highly permissive to a broad range of HIV [2,3]. All Env+ pseudoviruses mediated high levels of infection of HeLa TZM-bl cells. However, the T/F/acute Envs and some of the late stage non-mac-tropic Envs mediated significantly lower levels of TZM-bl infection compared to mac-tropic Envs (Figure 1B). These Envs may be less functional (i.e. they need more Env to achieve the same level of infectivity as other Envs) or they may assemble less efficiently onto pseudovirions. Either situation could result in underestimating their ability to mediate macrophage infection. To correct for this, we related Env+ pseudovirus infectivity for macrophages to TZM-bl infectivity by plotting macrophage infectivity titers as a percent of TZM-bl titers (Figure 1C). This approach suggests that T/F/acute Envs are not as tightly non-mac-tropic as late disease immune tissue Envs, yet are still significantly less mac-tropic compared to the highly mac-tropic Envs.

### Infection of CD4+ T-cells

**Figure 2:**
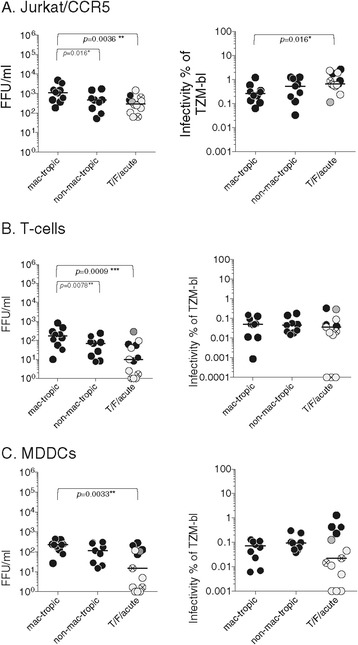
**Infection of Jurkat/CCR5, primary CD4+ T-cells and primary MDDCs by pseudovirions carrying mac-tropic and non-mac-tropic R5 envelopes.** Env+ GFP-reporter pseudovirus infection of Jurkat/CCR5 **(A)**, primary CD4+ T-cells **(B)** and LPS matured MDDCs **(C)**. Left panels shows FFU measured as GFP+ cells per ml of input virus. Right panels show infectivity as a percent of TZM-bl infectivity. Significant differences were evaluated using Mann Whitney or Wilcoxon matched pair tests as described in Methods. Only the p values that were significant are shown. Symbol colour designations are the same as described in Figure 1. Please also refer to Additional file 1: Figure S3, where FFU/ml for each Env+ pseudovirus is presented in bar graphs with standard deviation bars shown, while the column scatter plot of infectivity as a percent of TZM-bl infectivity is presented with each point labeled for Env used. Median values are marked.

Non-mac-tropic R5 variants are predominant in immune tissue, where CD4+ T-cells expressing high levels of CD4 are the major target cells [2,3,23]. We tested whether the T/F, acute and late non-mac-tropic R5 Envs have an increased ability to confer infection of CD4+ T-cells compared to highly mac-tropic R5 Envs. We first evaluated infection for Jurkat/CCR5 cells (Figure 2A, left panel and Additional file 1: Figure S3A). All Env + pseudoviruses mediated infection, although levels were low, varying from 0.062-2.65% of those recorded on TZM-bl cells. The mac-tropic R5 Envs consistently mediated significantly higher infection of Jurkat/CCR5 cells than both non-mac-tropic T/F/acute (*p* = 0.0036) and late disease (*p* = 0.016) Envs (Figure 2A).

We next tested infection on primary CD4+ T-cells. PBMCs obtained from at least two separate donors were stimulated with PHA and IL-2 before enriching for CD4+ T-cells via negative selection (Stemcell Tech. Inc.) and tested for infection using the GFP reporter pseudovirions. Both mac-tropic and non-mac-tropic R5 Envs conferred infection of CD4+ T-cells. However, infection for all Env+ pseudoviruses was surprisingly very low (Figure 2B, left panel and and Additional file 1: Figure S3B) with infectivity titers ranging from 0 – 0.33% of those recorded for HeLa TZM-bl cells. Neither do CD4 or CCR5 levels explain the low overall infection levels observed for Jurkat/CCR5 and primary CD4+ T-cells. Jurkat/CCR5 cells express low to high levels of CD4 and high CCR5, while primary CD4+ cells express high CD4 and variable CCR5 (data not shown and [35,36]).

Similar to Jurkat/R5 T-cell infectivity, primary T-cell infectivity for mac-tropic R5 Envs was significantly higher compared to non-mac-tropic, late disease R5 Envs (*p* = 0.0078) and compared to T/F/acute Envs (*p* = 0.0009).

As described above, T/F/acute and some late disease non-mac-tropic Envs mediated lower levels of infection for TZM-bl cells (compared to highly mac-tropic Envs), suggesting that they are less functional or carry lower levels of Env in pseudovirion preparations. To correct for different Env+ pseudovirus infectivities (measured on TZM-bl cells), we plotted T-cell infectivity as a percentage of the infectivity titers measured on TZM-bl cells. For both Jurkat/CCR5 and primary T-cells, values were similar for late disease mac-tropic and non-mac-tropic Envs (Figure 2A, B right panels and Additional file 1: Figure S3A-B), although T/F/acute Envs were higher for Jurkat/CCR5 cells and this just reached significance (*p* = 0.0158). CD4+ T-cell infectivity plotted as a percent of TZM-bl infectivity, was not significantly different among the 3 Env groups (Figure 2B, right panel). Taken together, none of this data convincingly supports a higher T-cell entry tropism for non-mac-tropic R5 Envs.

### Infection of MDDCs

We tested whether myeloid dendritic cells (mDCs) were susceptible to infection by mac-tropic or non-mac-tropic R5 Env+ pseudovirions. Myeloid DCs are derived from the same cell lineage as monocytes and macrophages and like these cell types, express low levels of CD4 [35]. Myeloid DCs express SAMHD1 [37,38] and sometimes APOBEC3G [39-41], restriction factors with the potential to restrict HIV replication. The low levels of CD4 and the presence of restriction factors has led to a consensus view that these cells are relatively insensitive to HIV-1 infection in vivo [42].

We prepared mDCs from blood monocytes as monocyte-derived dendritic cells (MDDCs) using standard (IL-4, GM-CSF) differentiation protocols [43,44] followed by maturation by LPS (0.1 μ/ml) and negative selection via magnetic beads (StemCell Tech. Inc.). We tested their sensitivity to infection by the panel of GFP-reporter Env+ pseudovirions (Figure 2C left panel and Additional file 1: Figure S3C). MDDCs were poorly susceptible to Env + pseudoviruses (Figure 2C, left panel), with highly mac-tropic Envs mediating detectable infection more consistently than non-mac-tropic T/F/acute Envs. MDDC infectivity plotted as a percent of TZM-bl infectivity, was variable but not significantly different among the 3 Env groups (Figure 2C, right panel).

### DEAE dextran and spinoculation rescue infection of CD4+ T-cells but not MDDCs

**Figure 3:**
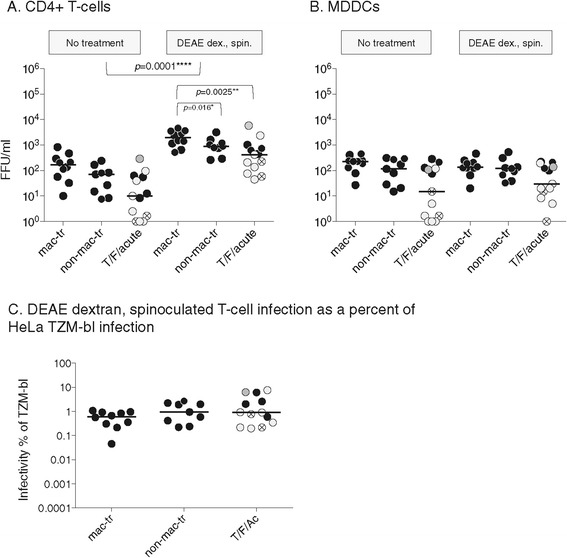
**DEAE dextran and spinoculation enhance infection for CD4+ T-cells but not for MDDCs.** Infection in the presence and absence of DEAE dextran and spinoculation on CD4+ T-cells **(A)** and MDDCs **(B)**. DEAE dextran and spinoculation enhance non-mac-tropic Envs and mac-tropic Envs to similar extents **(C)**. Significant differences were evaluated using Mann Whitney or Wilcoxon matched pair tests as described in Materials and Methods. Figure 3 experiments were done in conjunction with the direct infections shown in Figure 2. Figure 3 data for infection without DEAE dextran and spinoculation is therefore the same as that shown in Figure 2. Only the p values relevant for DEAE dextran and spinoculation that show significance are shown. Symbol colour designations are the same as described in Figure 1. Please also refer to Additional file 1: Figure S4, where FFU/ml for each Env+ pseudovirus is presented in a bar graph with standard deviation bars shown, while the column scatter plot of infectivity as a percent of TZM-bl infectivity is presented with each point labeled for Env used. Median values are marked.

T/F and non-mac-tropic Envs bind CD4 less efficiently than mac-tropic Envs [2,3,11,26] and it is possible that such Envs mediate less efficient binding of virions to CD4+ T-cells. We reasoned that this could be revealed by preferential enhancement of T-cell infection by non-mac-tropic Envs (compared to mac-tropic Envs) following DEAE dextran and spinoculation mediated attachment of virions. In parallel with the direct infectivity assays shown in Figure 2, we tested if infection of CD4+ T-cells could be facilitated by increasing attachment of virus particles using DEAE dextran (DD) and spinoculation and compared with MDDC infection. Our data showed a significant enhancement of infection for CD4+ T-cells by all Env+ pseudovirions by an average of >10-fold, with the three Env+ virus groups enhanced to similar extents (Figure 3A, C). Importantly, this data is consistent with non-mac-tropic Envs (including T/F Envs) binding to T-cells with similar efficiencies as mac-tropic variants. In contrast to T-cells, DD and spinoculation had little effect on the low levels of infection detected for MDDCs (Figure 3B).

Mac-tropic R5 Envs mediated the highest infection levels for T-cells in the presence of DD and spinoculation (Figure 3A), with infection significantly higher than T/F/acute Envs (*p* = 0.0025) and late disease non-mac-tropic (*p* = 0.016) R5 Envs. Plotting infectivity titers as a percent of HeLa TZM-bl indicated that DD and spinoculation facilitated T-cell infection by mac-tropic and non-mac-tropic Envs from early and late in infection with similar efficiencies (Figure 3C and Additional file 1: Figure S4).

### MDDC-mediated trans-infection of T-cells

**Figure 4:**
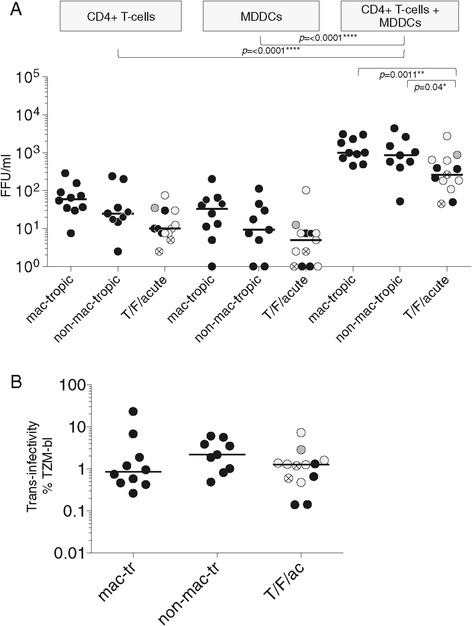
**MDDC mediated trans-infection of CD4+ T-cells by Env+ pseudovirions. (A)** Trans-infection of CD4+ T-cells following virion capture by MDDCs is substantially more efficient than cell free infection of T-cells or MDDCs alone. T/F/acute and late disease non-mac- and mac-tropic R5 Env+ pseudovirions are all enhanced. Note that for late disease non-mac-tropic and mac-tropic R5 Envs, trans-infectivity titers are not significantly different. Infectivities are shown as the number of FFU/ml (GFP+ cells per ml) of input virus. **(B)** Infectivity as a percent of infection for TZM-bl cells suggests that trans-infection preferentially enhances non-mac-tropic R5 Env+ pseudovirions, although differences are not significant. Symbol colour designations are the same as described in Figure 1. Please also refer to Additional file 1: Figure S5, where FFU/ml for each Env+ pseudovirus is presented in a bar graph with standard deviation bars shown, while the column scatter plot of infectivity as a percent of TZM-bl infectivity is presented with each point labeled for Env used. Median values are marked.

DCs capture HIV-1 particles and present them to CD4+ T-cells via synapses. This process bypasses the requirement for cell-free virions to attach directly to T-cells. However, presented virions still need to bind CD4 and CCR5, which are concentrated on the T-cell side of the synapse, for fusion and entry to be triggered. This trans-infection process greatly increases the efficiency of CD4+ T-cell infection [45-47]. We evaluated whether non-mac-tropic Envs that target T-cells in vivo had evolved more efficient entry via trans-infection compared to mac-tropic Envs. Both immature and mature DCs are capable of eliciting trans-infection of CD4+ T-cells [45,47]. However, mature DCs are the most efficient for this process [48-50]. We therefore tested whether LPS-matured MDDCs first treated with Env+ pseudovirions could confer efficient infection of autologous CD4+ T-cells. In the direct infectivity assays for MDDCs and CD4+ T-cells described above, target cells had been treated with Env+ pseudoviruses for three hours before adding growth medium without washing. For the trans-infection assays, we limited MDDC exposure to virus to 1 hour, before washing and co-culturing with autologous PHA, IL-2 stimulated CD4+ T-cells. In parallel, MDDCs and CD4+ T-cells were treated separately with Env+ pseudovirions for one hour before washing and incubating. Direct infection of MDDCs and CD4+ T-cells using this approach was very low and variable (Figure 4A). Trans-infection via MDDCs resulted in substantially higher levels (>10-fold) of CD4+ T-cell infection compared to direct infection of T-cells or MDDCs alone (Figure 4A).

Trans-infection of CD4+ T-cells by late stage non-mac-tropic Envs was not significantly different compared to mac-tropic Envs (Figure 4A), contrasting with significant differences detected for TZM-bl, macrophage, Jurkat/CD4 and primary T-cell infection shown in Figures 1, 2 and 3. This result suggests that trans-infection preferentially enhances late disease non-mac-tropic Envs over mac-tropic Envs. However, while trans-infection greatly enhanced T-cell infection for T/F/acute Envs, infectivity was still significantly lower compared to the highly mac-tropic R5 Envs (*p* = 0.04).

We next plotted MDDC/T-cell infectivities as a percent of those measured on the highly susceptible HeLa TZM-bl cells to correct for possible differences in the efficiency of pseudotype production from 293 T cells. Figure 4B suggests that late stage non-mac-tropic R5 Env+ pseudoviruses are enhanced to a significantly greater extent than mac-tropic pseudovirions, although this was not significant.

These experiments suggest that trans-infection via MDDCs preferentially enhances infection mediated by non-mac-tropic R5 Envs from late disease over highly mac-tropic Envs. However, trans-infection did not confer higher T-cell infection for late disease non-mac-tropic R5 Envs and did not fully rescue T/F/acute stage Envs. Nevertheless, these observations emphasize the role cell: cell transfer of virus must play during transmission and viral replication in immune tissue particularly for non-mac-tropic Envs.

## Discussion

We investigated T-cell tropism of non-mac-tropic R5 Envs that require high levels of CD4 for infection including several T/F and acute stage Envs and compared to highly mac-tropic variants mostly derived from brain tissue. Viruses carrying non-mac-tropic R5 Envs predominate in immune tissue where they presumably carry a selective advantage over, and outcompete more mac-tropic R5 variants that evolve. We wanted to evaluate whether their predominance was due to the evolution of an enhanced ability of the envelope glycoproteins to mediate infection of CD4+ T-cells. However, none of the data presented indicates that non-mac-tropic Envs consistently mediate more efficient infection of CD4+ T-cells compared to mac-tropic Envs.

A limitation of our study is that we focused on Env and viral entry rather than full length viral clones and full replication. Viral entry includes attachment, receptor interactions, fusion etc., and is likely to be an important determinant of tropism in vivo, with adaptation for CD4, CCR5 binding and escape from neutralizing antibodies potential responses to selective pressures in vivo. Nevertheless, it is possible that non-mac-tropic Envs mediate enhanced replication in the context of multiple replication cycles that include Env expression and assembly.

We took two approaches to compare the infectivity of mac-tropic and non-mac-tropic R5 Env+ pseudoviruses. First, we titrated different Env+ pseudoviruses on each cell type and compared their infectivity. This approach showed that pseudoviruses carrying mac-tropic Envs mediated significantly higher levels of infection for macrophages, Jurkat/CCR5 and primary T-cells compared to non-mac-tropic Envs. Only when T-cells were trans-infected via MDDCs, did late disease non-mac-tropic Envs mediate similar levels of infection compared to mac-tropic Envs suggesting that this route of infection may preferentially support viruses carrying non-mac-tropic Envs.

One concern is that different Env+ pseudovirus preparations generated in 293 T cells may vary in the amount of infectious virus produced, which is influenced by variation in transfection efficiency, levels of Env assembled and differences in the functionality of each Env. So, in a second approach, we standardized our Env+ pseudovirus infectivity data by plotting infectivity titers as a percent of that measured on highly permissive HeLa TZM-bl cell line as we previously reported [2,3]. All Env+ pseudoviruses mediated respectable infectivity titers on HeLa TZM-bl cells (Figure 1B). However, the lower titers for several late disease non-mac-tropic Envs and particularly for T/F/acute Envs could in part explain their lower levels of infectivity for the other cell types types tested here. Plotting infectivity as a percent of TZM-bl infectivity corrects for differences in overall pseudovirus infectivity and showed that non-mac-tropic Envs mediated similar levels of infectivity for Jurkat/CCR5 and primary T-cells compared to mac-tropic Envs. However, this approach still did not provide evidence to suggest that non-mac-tropic Envs were more T-cell tropic compared to mac-tropic Envs.

Using HeLa TZM-bl titers to normalize Env+ pseudovirus infectivity is a useful approach. However, it does not discern whether decreased titers for non-mac-tropic Env+ pseudoviuses could be due to Envs being less functional or expressed less efficiently. We speculate that non-mac-tropic viruses replicating in immune tissue may evolve Envs that are tightly closed to protect against neutralizing antibodies. Such Envs may be less functional compared to mac-tropic Envs from the brain i.e. they need more Env to achieve the same level of infectivity as mac-tropic Envs. Envs that are inherently less functional will result in less infectious virions in vivo as well as in vitro. We tried to evaluate whether different Env+ pseudovirus preparations varied in the amount of gp120 present or whether some Envs were less functional. To evaluate gp120 concentrations in Env+ pseudovirus preparations, we used a commercial ELISA assay to measure gp120 levels present in Env+ pseudovirus preparations as an overall measure of Env expression. However, this approach was only partially successful due to variation in the sensitivity in detection of gp120s from different clade B and particularly non-clade B Envs. However, we were able to investigate gp120 levels in pseudotype preparations carrying clade B Envs from several of the late disease subjects. This additional data provides evidence that gp120 levels in pseudoviruses of non-mac-tropic Envs are slightly lower for some subjects, but also suggests that several non-mac-tropic Envs are less functional than mac-tropic Envs from the same subject (Additional Data text and Additional file 1: Figure S6). These data suggest that at least some non-mac-tropic Envs are less functional than highly mac-tropic Envs, but not more tropic for T-cell entry.

Env+ pseudovirus infection of primary CD4+ T-cells with cell-free virus was surprisingly low for all Envs. However T-cell infection was greatly increased by facilitating attachment and perhaps cell activation using DEAE dextran and spinoculation [51]. This observation is consistent with a defect in virion binding, which is overcome either by DEAE dextran and spinoculation or via MDDC capture of viral particles and trans-infection of CD4+ T-cells. We also used enhancement of infection by DEAE dextran and spinoculation to explore whether the different virus groups bound to CD4+ T-cells differently. Mac-tropic Envs bind CD4 much more efficiently compared to T/F and other non-mac-tropic R5 viruses and might be expected to preferentially bind to CD4+ T-cells. Alternatively, it is possible that non-mac-tropic viruses (that replicate in T-cells in immune tissue) may be able to bind to T-cells effectively even without a strong Env: CD4 interaction. We couldn’t use direct virus binding assays to test this as it is not possible to distinguish between infectious and non-infectious virions (with the latter being the most abundant). However, we reasoned that differences in binding of infectious virions to CD4+ T-cells could be revealed by enhancing attachment with DEAE dextran and spinoculation. For example, DEAE dextran and spinoculation might be expected to preferentially aid attachment to CD4+ T-cells by T/F and non-mac-tropic Env+ viruses, which bind CD4 with low efficiency compared to mac-tropic Envs. Importantly, our data indicates that this is not the case and that non-mac-tropic Envs (including T/F Envs) bind to T-cells with similar efficiency as mac-tropic variants.

Trans-infection following capture of virions by MDDCs helped all Env+ viruses overcome the deficit of cell-free virus binding to T-cells. After attachment to MDDCs, viruses are then targeted to DC:T-cell synapses where CD4 and CCR5 (highly concentrated on the T-cell side [49]) trigger fusion and entry. This environment preferentially enhanced infection of late disease non-mac-tropic Env+ viruses, resulting in levels of T-cell infection that were not significantly different compared to mac-tropic Env+ viruses. The mechanistic basis behind preferential enhancement of non-mac-tropic Env+ viruses is unclear. However, it is likely that the concentration of CD4 on the T-cell side of the synapse plays a major role, enabling non-mac-tropic Env+ virions to bind several CD4 molecules to increase avidity of attachment. This process may help such Envs overcome a low affinity for CD4 to trigger fusion. It is also important to point out that we did not study the transfer of HIV particles from an infected T-cell to uninfected T-cells via virological synapses i.e. cis-infection [52]. Nevertheless, our observations support an important role for trans-infection and synapse dependent entry in the replication of HIV in immune tissue and for late disease non-mac-tropic viruses in particular.

T/F viruses and those replicating in the acute stage of infection are believed to predominantly target T-cells [53-55]. Some studies support an enhanced fitness for T/F viruses [56,57], although others failed to confirm that T/F Env genes mediated higher infection of T-cells [58,59]. Here, we studied a diverse panel of T/F and acute stage Envs for T-cell infection. T/F/acute Envs generally yielded pseudovirions with lower infectivity (compared to late disease Envs) for the different cell types tested. Several of the T/F/acute Envs were from clade A and C, with one an A/C recombinant. Env+ pseudoviruses were made using a clade B env-minus pNL4.3 clone that provided all viral proteins except Env to form virus particles. While all Envs yielded Env+ pseudoviruses with respectable infectivity titers on HeLa TZM-bl cells, we can’t rule out the possibility that lower TZM-bl infectivity titers for some of the non-clade B Envs was due to reduced Env assembly on to clade B viral cores.

Our data do not support the evolution of Envs that preferentially mediate T-cell entry over mac-tropic variants. The requirement of non-mac-tropic R5 Envs for high CD4 levels to induce infection is likely to be a major factor in limiting their replication to T-cells and excluding them from cells expressing lower CD4 levels, rather than an enhanced and Env-determined specific tropism for T-cell entry that precludes macrophage infection. While we believe that neutralizing antibodies may be a major selective force in immune tissue following their generation, it remains unclear why the non-mac-tropic phenotype is maintained during the acute stage of replication before sero-conversion when HIV specific antibodies are absent. An important conclusion from our results is therefore that there must be a powerful unknown force during transmission, the acute phase and in immune tissue throughout disease, that selects against envelopes that can mediate macrophage infection so that variants with non-mac-tropic Envs are the fittest and prevail.

## Conclusions

We demonstrate that T/F, early or late disease non-mac-tropic R5 Envs do not confer an enhanced T-cell entry tropism compared to mac-tropic Envs. We show that virion capture by mDCs and subsequent trans-infection of T-cells via synapses is important for optimal infection by non-mac-tropic R5 Env+ viruses, enhancing infectivity to levels equivalent to mac-tropic viruses, but not higher. We conclude that non-macrophage-tropism of HIV-1 R5 Envs is determined in part by an adaptation to an unknown environmental pressure that constrains mac-tropic variants.

## Methods

### Envelope clones

We investigated 33 R5 envelopes previously characterized as highly mac-tropic or non-mac-tropic (Table 1) [2,3,23,60]. Mac-tropic and non-mac-tropic R5 envelopes from seven AIDS patients were included. We used at least one highly mac-tropic and one non-mac-tropic Env+ from each individual. For subject NA20, we investigated a second highly mac-tropic Env and for CA110, we included two highly mac-tropic R5 Envs, one from brain and one from spleen alongside two non-mac-tropic Envs from spleen. All these envelopes had previously been highly characterized and their genotypes and phenotypes described [2,3,23,61]. We also tested mac-tropic JR-FL and non-mac-tropic JR-CSF, which were derived from the same individual and have been used extensively in HIV research [4].

**Table 1:** **T/F, acute, late stage non-mac-tropic and late stage mac-tropic R5 Envs**

**Patient**	**Clade**	**Non-mac-tropic**			**Mac-tropic**			**Reference**
		**Env**	**Origin**	**Full ID**	**Env**	**Origin**	**Full ID**	
**AIDS patients**								
NA20	B	LN8	LN^1^		B59	FL^2^		[3]
					B501	FL		
NA420	B	LN40	LN		B33	FL		[3]
JR	B	JR-CSF	CSF^3^		JR-FL	FL		[4]
P1114	B	98-27	Plasma		98-15	Plasma		[3]
P7766	B	SP1	Spleen	SP13-33-41	FL2	FL	FL19-56-66	[23]
P10017	B	SP2	Spleen	SP10-9-65	FL1	FL	FL9-1-2	[23]
P6568	B	SP1	Spleen	SP6-11-9	FL1	FL	FL11-1-249	[23]
CA110	B	SP2	Spleen	SP53-23-131	OC1	OL^4^	OC58-11-57	[23]
		SP3 SP4	Spleen Spleen	SP53-6-122 SP52-16-50	SP1	Spleen	SP52-13-34	
**T/F**								
3 T	B	3 T	Plasma	p1054.TC4.1499				[60]
6 T	B	6 T	Plasma	p63358.p3.4013				[60]
15 T	B	15 T	Plasma	p700010040.C9.4520				[60]
19 T	B	19 T	Plasma	pPRB958_06.TB1.4305				[60]
**Acute Stage**								
R463F	A1	R463F	Plasma	R463FPL16MAR07EnvE44				[62]
R880F	A1	R880F	Plasma	R880FPL12JAN07EnvA6				[63]
Z185F	C	Z185F	Blood	Z185FPB24AUG02ENV3.1				[63,64]
Z205F	C	Z205F	Blood	Z205FPB27MAR03ENV1.1				[63,64]
R66M	A/C	R66M	Plasma	R66MPL7MAR06.3A9Env				[63]
Z1792M	C	Z1792M	Plasma	Z1792MPL18DEC07.3G7Env2				[63]
Z201M	C	Z201M	Plasma	Z201MPL7FEB03ENV2.1				[13]
Z221M	C	Z221M	Plasma	Z221MPL7MAR03ENV2.1				[13]
Z153M	C	Z153M	Plasma	Z153MPL13MAR02ENV5.1				[13]

We selected pairs of highly mac-tropic and non-mac-tropic R5 Envs from 8 subtype B infected AIDS patients (including JR-FL and JR-CSF) for study. For subject NA20, we included 2 highly mac-tropic Envs. For CA110, we included two highly mac-tropic Envs and two non-mac-tropic spleen Envs. Except for JR-FL and JR-CSF, all Envs had been PCR amplified directly from patient tissues. We also included 13 diverse R5 Envs previously reported as T/F or isolated from the acute stage of infection. In this group, we included Envs from different subtypes. 1. Lymph node; 2. Frontal lobe; 3. Cerebral spinal fluid; 4. Occipital lobe.

For comparison with these late disease envelopes, we included 13 envelopes that were derived from early in infection. Sexually transmitted viruses were reported to carry fewer glycans [65-71] and shorter variable loops [72,73] that may confer an enhanced fitness [56], although this is less clear for clade B viruses [68,70,74,75]. These early viruses predominantly target T-cells [53-55] and are thus the most likely to be highly adapted for T-cells. We selected a diverse range of envelopes from different clades to provide the best chance of establishing whether we could detect any early stage Envs that were more efficient than late disease mac-tropic Envs for infection of CD4+ T-cells. We included ‘transmitter/founder’ (T/F) envs constructed from the consensus sequences of early, replicating virus [60] as well as some from the acute stage of replication. These included four clade B ‘transmitter/founder’ (T/F) envelopes and 9 clade A, AC and C envs derived from the acute stages of infection [62,63,66,76]. All envs were expressed from Rev-Env expression vectors, which confer efficient Env expression and the formation of Env+ pseudovirions. Most envs were expressed from pcDNA3.1TOPO. Envs from subjects NA20, NA420, P1114 and JR were expressed from pSVIIIenv. Both vectors have been shown to support the production Env+ pseudovirions with equivalent levels of infectivity (not shown).

### Cell cultures

293 T cells were used to prepare Env-containing (Env+) pseudovirions by transfection. Env+ pseudovirions were first titrated on HeLa TZM-bl cells [77]. 293 T and HeLa TZM-bl cells were cultured in Dulbecco modified Eagle medium (DMEM) with 4% fetal bovine serum (FBS) and gentamicin (10 μg/mL).

Jurkat E6-1 cells stably expressing CCR5 were obtained from H. Gottlinger. CCR5 had previously been introduced using pCXbst-CCR5 vector. These cells were cultured in 10% FBS in RPMI with 5 μg/ml blasticidin.

Primary macrophages were prepared from elutriated blood monocytes. Briefly, 0.5 ml of elutriated monocytes (5x10^5^/ml) was plated in each well of 48-well cell culture dishes and cultured in 10% human AB+ plasma in DMEM for 5 to 7 days before infection. Alternatively, 5x10^7^ peripheral blood mononuclear cells (PBMC) from a buffy coat (Research Blood Components LLC, Boston, MA) were plated into 14-cm bacterial culture dishes for 3 h before extensively washing away non-adherent cells, culturing overnight, and repeating the washes. The adhered monocytes were then cultured for 5 to 7 days in 10% AB+ human plasma in DMEM before treatment with EDTA and transfer to 48-well tissue culture dishes the day prior to infection [2,3].

MDDCs were prepared from blood monocytes by adherence using standard procedures [44]. They were cultured in DMEM+ 10% fetal calf serum containing 100 ng/mL granulocyte-macrophage colony stimulating factor (GM-CSF) and 40 ng/mL IL-4 (Peprotech Inc.) for 5 days. MDDCs were then activated with LPS (100 ng/ml) for 48 hours before infection [44]. The day before infection, MDDCs were treated with EDTA for 7 minutes at 37°C, before gently scraping from the flask, undergoing DC negative selection to remove any contaminating cells using EasySep (Stemcell Technologies) and plating into 96 well plates for assays. Activated MDDCs expressed CD11c and showed high expression of CD83, markers for DCs and activated DCs respectively (not shown).

Primary PBMCs were isolated from whole human blood or buffy coats by Ficoll-Paque separation. Cells were washed twice with sterile PBS and cultured in RPMI medium with 10% fetal bovine serum (FBS), 5 μg/ml phytohemagglutinin and after two days, with IL-2 (5 ng/mL, Roche Inc.). CD4+ T-cells were isolated from stimulated PBMCs using negative selection for CD4+ T-cells (Stemcell Technologies).

### Preparation of Env+ pseudoviruses

1.25 μg of Env+ plasmids carrying *rev-env* sequences were co-transfected with 1.25 μg of pNL43 that carried a premature stop codon in the envelope gene and 0.625 μg of pHIvec2-GFP plasmid [78] into 293T cells using calcium phosphate (Profection mammalian transfection kit, Promega Inc.) [2]. The cell supernatant was changed 8-18 hrs post-transfection (4% FBS DMEM). Pseudovirions were harvested 48 h post-transfection, clarified by low-speed centrifugation, aliquoted into 0.5 ml portions, and snap-frozen in liquid nitrogen.

### Infectivity assays

We used Env+ pseudovirions carrying a GFP reporter gene to investigate infection of different cell types. Following infection of cells, GFP is expressed from a reporter gene so that only infected cells become GFP+ (Additional file 1: Figure S1A-D). This system allows infected GFP+ cells to be observed using fluorescent microscopy and this was particularly important in trans-infection assays where infected T-cells can easily be distinguished from MDDCs. All Env+ pseudoviruses were titrated on each cell type, with undiluted and tenfold dilutions of virus supernatant added. Infectivities were expressed as FFU/ml with each GFP+, infected cell representing an individual focus of infectivity. Infectivity was also standardized to titers measured on HeLa TZM-bl cells and plotted as infectivities as a percent of TZM-bl infectivity. Infectivity titers were usually calculated from wells containing 10-150 GFP+ cells per well. We confirmed that estimates of infectivity for primary T-cells (as percentages of HeLa TZM-bl FFUs) stayed the same over a range of 2-fold dilutions of Env+ pseudoviruses where 10-150 FFUs were counted. In this experiment, estimates of infectivity (as percent of TZM-bl titers) varied by less than 1.5-fold across at least 4 two-fold dilutions (Additional file 1: Figure S2). This approach allowed us to measure maximal infectivity for each Env+ pseudovirus on specific cell targets as well as relating infectivity to that measured on the highly permissive TZM-bl cell line. Infectivity data for the different cell types was averaged from two independent experiments. For primary cells, at least two independent experiments were done on cells derived from different donors. Infection details for specific cell types are described below.

HeLa TZM-bl cells were plated at 0.5 ml per well (5x10^5^ cells/ml) in 48-well dishes the day prior to infection and infected with Env+ pseudovirions carrying a GFP reporter gene. After 72 h, GFP+ FFU were quantified by microscopy [2].

Macrophages seeded in 48 well plates were pretreated with 0.1 ml DEAE dextran (10 μg/ml) in DMEM medium containing 10% human plasma for 30 min at 37°C before Env+ pseudoviruses carrying a GFP reporter gene were added and spinoculating plates for 45 minutes in a benchtop centrifuge [51]. Infected macrophages were incubated for a further 3 h at 37°C before the addition of 0.4 ml of DMEM (10% FBS) and incubating at 37°C for seven days. DEAE dextran and spinoculation enhance virus infectivity by approximately 20-fold by increasing attachment [51] and entry [79]. Infection following this procedure does not bypass the requirement of CD4 and CCR5 for infection, which remains sensitive to entry inhibitors including maraviroc (not shown). Env^+^ pseudovirions are capable of only a single round of replication so that focus-forming units (FFU) were estimated 5-7 days post-infection by counting individual GFP+ cells by fluorescent microscopy.

CD4+ T-cells, MDDCs and Jurkat clones were infected with GFP reporter Env+ pseudovirus in 96-well plates (1.3 × 10^5^ cells/well). CD4+ T-cells and MDDCs were infected in the presence and absence of DEAE dextran and spinoculation as described above. 100 μL of viral supernatants were added to wells, and infection quantified 2 days post infection by counting GFP+ cells using a fluorescent microscope. Env+ pseudovirus infectivities were plotted directly and also as a percent of infectivity measured in HeLa TZM-bl cells.

### Trans-infection assay

IL-4, GM-CSF and LPS (100 ng/ml) activated MDDCs were treated with EDTA, scraped, enriched by negative selection and aliquoted into 96-well V bottom plates (9 × 10^4^ cells/well) the day before assay. 100 μL of Env+ pseudovirus supernatant was added, and cells incubated for 1 hr at 37°C. Cells were then washed three times with PBS, and co-cultured with autologous CD4+ T-cells prepared as described above. Cells in each well were then transferred into 96-well flat bottom cell culture plates. Infectivity was quantified 2 days post infection by counting GFP+ T-cells. GFP+ T-cells were readily distinguished from MDDCs by their small rounded morphology. In trans-infection experiments, the vast majority of GFP+ cells were T-cells. CD4+ T-cells and MDDCs were also infected individually in parallel with the MDDC/T-cell cocultivations, before washing after 1 hour and incubating as described above.

### ELISA measurements of gp120 and p24

The concentration of gp120 in Env+ pseudovirion preparations was measured using an HIV-1 gp120 antigen capture assay (Advanced Bioscience Laboratories Inc.), while p24 levels were measured using an HIV-1 p24 antigen capture assay (Advanced Bioscience Laboratories Inc.). Assays were performed using unpurified 293T supernatants containing pseudoviruses.

### Statistics

Significant differences between values for different Env groups were evaluated using Mann Whitney or Wilcoxon matched pair tests using Prism 6 for Mac OS X. Wilcoxon matched paired tests were used to compare Envs from late disease where each pair of Envs was derived from an individual subject. Mann Whitney tests were used to compare unrelated T/F/acute Envs with late disease Envs. For some subjects, more than one mac-tropic or non-mac-tropic Env was derived from an individual. In this situation, values were averaged so that only one value per Env group per subject was analyzed.

## Additional file

Additional file 1:
**Additional data and figures.** **Figure S1.** Infection of cells using a GFP reporter+ Env+ pseudovirus. (A) HeLa TZM-bl; (B) primary CD4+ T-cells; (C) CD4+ T-cells infected following MDDC capture of virions and trans-infection. Note a GFP+ T-cell adjacent to a clump of MDDCs, which also contains additional GFP+ cells that are out of the plane of focus; (D) infected MDDC; and (E) Low level infection of MDDCs is inhibited by AZT. Note: please view electronic version of panels A-D. **Figure S2.** Estimation of Env+ pseudovirus infectivity for CD4+ T-cells as a percent of that measured on HeLa TZM-bl. Left panel; FFU counts from infection of primary T-cells using 2-fold dilutions of Env+ pseudovirus preparations. Right panel; Estimation of infectivity as a percent of TZM-bl using FFU counts. **Figure S3.** Env+ pseudovirus infectivity for Jurkat/CCR5 (A), primary T-cells (B) and MDDCs (C). For each cell type, infectivity is plotted as FFU/ml of input virus with standard deviations shown (top panels), Env+ pseudovirus infectivities as percentages of that recorded on HeLa TZM-bl are also shown as labeled points in a column scatter plot (bottom panels). Symbol colour designations are the same as described in Figure 1. **Figure S4.** Env+ pseudovirus infectivity for CD4+ T-cells following DEAE dextran and spinoculation. See Figure S3 for more details. **Figure S5.** Env+ pseudovirus infectivity for CD4+ T-cells following MDDC capture and trans-infection. See Figure S3 for more details. **Figure S6.** (A) Gp120 and p24 concentrations in Env+ pseudovirus preparations of late stage Envs of 6 individuals. (B) HeLa TZM-bl infectivity plotted as a ratio with gp120 (left) or p24 (right) concentrations. TZM-bl/gp120 ratios (shown in B, left lanel) indicate that non-mac-tropic Envs from 4 of 6 individuals are less functional compared to mac-tropic Envs (i.e. they need more Env to achieve the same level of infectivity as their mac-tropic counterparts).
