# Supplementary material for: HIV-1 non-macrophage-tropic R5 envelope glycoproteins are not more tropic for entry into primary CD4+ T-cells than envelopes highly adapted for macrophages
Source: Retrovirology. 2015 Mar 14;12:25. doi: 10.1186/s12977-015-0141-0 (PMC4373511; doi:10.1186/s12977-015-0141-0)
Supplement: Additional file 1: — Additional data and figures. Figure S1. Infection of cells using a GFP reporter+ Env+ pseudovirus. (A) HeLa TZM-bl; (B) primary CD4+ T-cells; (C) CD4+ T-cells infected following MDDC capture of virions and trans-infection. Note a GFP+ T-cell adjacent to a clump of MDDCs, which also contains additional GFP+ cells that are out of the plane of focus; (D) infected MDDC; and (E) Low level infection of MDDCs is inhibited by AZT. Note: please view electronic version of panels A-D. Figure S2. Estimation of Env+ pseudovirus infectivity for CD4+ T-cells as a percent of that measured on HeLa TZM-bl. Left panel; FFU counts from infection of primary T-cells using 2-fold dilutions of Env+ pseudovirus preparations. Right panel; Estimation of infectivity as a percent of TZM-bl using FFU counts. Figure S3. Env+ pseudovirus infectivity for Jurkat/CCR5 (A), primary T-cells (B) and MDDCs (C). For each cell type, infectivity is plotted as FFU/ml of input virus with standard deviations shown (top panels), Env+ pseudovirus infectivities as percentages of that recorded on HeLa TZM-bl are also shown as labeled points in a column scatter plot (bottom panels). Symbol colour designations are the same as described in Figure 1. Figure S4. Env+ pseudovirus infectivity for CD4+ T-cells following DEAE dextran and spinoculation. See Figure S3 for more details. Figure S5. Env+ pseudovirus infectivity for CD4+ T-cells following MDDC capture and trans-infection. See Figure S3 for more details. Figure S6. (A) Gp120 and p24 concentrations in Env+ pseudovirus preparations of late stage Envs of 6 individuals. (B) HeLa TZM-bl infectivity plotted as a ratio with gp120 (left) or p24 (right) concentrations. TZM-bl/gp120 ratios (shown in B, left lanel) indicate that non-mac-tropic Envs from 4 of 6 individuals are less functional compared to mac-tropic Envs (i.e. they need more Env to achieve the same level of infectivity as their mac-tropic counterparts). [file 12977_2015_141_MOESM1_ESM.zip › 1034352199146124_add1.pdf]

Additional file 1

Additional data and figures

Gp120, p24 and infectivity of clade B Env+ pseudovirion preparations

Different levels of Env+ pseudovirus infectivity could be due to differences in the amount of virus particles produced and the levels of Env assembled. To try to address this, we measured the levels of gp120 and p24 present in pseudovirion preparations to establish whether differences might contribute to differences in the infectivities observed. However, we were unable to identify an ELISA assay that reliably and accurately measured gp120s from different strains and clades. We used the Advanced Bioscience Laboratories gp120 ELISA, which is designed for detecting clade B gp120s but can detect gp120s from other clades. However, this assay resulted in ultra-low readings for gp120s from clade B infected subjects CA110 and 10017 (even though these Envs formed Env+ pseudoviruses with high titers for TZM-bl cells) and we were not confident that the gp120 measurements for these Envs were accurate. Nevertheless, we still believed it was worth evaluating gp120s from the remaining 6 clade B AIDS patients and further reasoned that closely related, paired gp120s from a single individual are likely to be detected with similar efficiencies. The results obtained using this approach are described in Additional fig. 6.

Gp120 concentrations ranged over 25-fold from 771 to 21,457 pg/ml, while p24 ranged 13-fold from 12,409 to 164, 087 pg/ml. Mac-tropic and non-mac-tropic Env+ pseudoviruses did not differ significantly in gp120 or p24 content. However, for most of the 6 subjects analyzed, gp120 levels for the mac-tropic R5 Env+ pseudoviruses were higher compared to non-mac-tropic Envs, while only two carried more p24 (Additional fig. 6A).

No correlations between gp120 or p24 concentrations and infectivity for HeLa TZM-bl cells were detected (data not shown). However, we plotted ratios of TZM-bl infectivities to gp120 and p24 concentrations (Additional fig. 6B). If differences in TZM-bl infectivity titers are simply due to differences in Env or p24 content, then TZM-bl: gp120 ratios for mac-tropic and non-mac-tropic Env pairs should be equivalent. However, Additional. fig. 6B shows that the TZM-bl: gp120 and TZM-bl: p24 ratios for mac-

tropic Envs from 4 of 6 and 3 of 6 subjects respectively were substantially higher than the corresponding non-mac-tropic Env and this is consistent with a higher functionality for mac-tropic Envs i.e. they need less Env to achieve the same level of infectivity as non-mac-tropic Envs from the same subjects. Together, these data suggest that the reduced TZM-bl infectivity recorded for several non-mac-tropic Envs is due in part to less Env but also in part due to reduced functionality. Although limited, this data provides some support for possibility that some of the non-mac-tropic Envs are less functional than highly mac-tropic Envs.

Additional figure legends

Additional Fig. 1. Infection of cells using a GFP reporter+ Env+ pseudovirus. (A) HeLa TZM-bl; (B) primary CD4+ T-cells; (C) CD4+ T-cells infected following MDDC capture of virions and trans-infection. Note a GFP+ T-cell adjacent to a clump of MDDCs, which also contains additional GFP+ cells that are out of the plane of focus; (D) infected MDDC; and (E) Low level infection of MDDCs is inhibited by AZT. Note: please view electronic version of panels A-D.

Additional Fig. 2. Estimation of Env+ pseudovirus infectivity for CD4+ T-cells as a percent of that measured on HeLa TZM-bl. Top panel; FFU counts from infection of primary T-cells using 2-fold dilutions of Env+ pseudovirus preparations. Bottom panel; Estimation of infectivity as a percent of TZM-bl using FFU counts. Infectivity titers varied by less than 1.5-fold across at least 4 two-fold dilutions. This experiment shows that measurements of infectivity as a percent of TZM-bl stay the same at different amounts of virus exposure. This verifies our approach to use % TZM-bl to compare infectivities of different Env+ pseudoviruses.

Additional Fig. 3. Env+ pseudovirus infectivity for Jurkat/CCR5 (A), primary T-cells (B) and MDDCs (C). For each cell type, infectivity is plotted as FFU/ml of input virus with standard deviations shown (top

panels), Env+ pseudovirus infectivities as percentages of that recorded on HeLa TZM-bl are also shown as labeled points in a column scatter plot (bottom panels). Symbol colour designations are the same as described in fig. 1.

Additional Fig. 4. Env+ pseudovirus infectivity for CD4+ T-cells following DEAE dextran and spinoculation. Infectivity is plotted as FFU/ml of input virus with standard deviations shown (top panels), Env+ pseudovirus infectivities as percentages of that recorded on HeLa TZM-bl are also shown as labeled points in a column scatter plot (bottom panel). Symbol colour designations are the same as described in fig. 1.

Additional Fig. 5. Env+ pseudovirus infectivity for CD4+ T-cells following MDDC capture and trans-infection. Infectivity is plotted as FFU/ml of input virus with standard deviations shown (top panel), Env+ pseudovirus infectivities as percentages of that recorded on HeLa TZM-bl are also shown as labeled points in a column scatter plot (bottom panel). Symbol colour designations are the same as described in fig. 1.

Additional Fig. 6. (A) Gp120 and p24 concentrations in Env+ pseudovirus preparations of late stage Envs of 6 individuals. (B) HeLa TZM-bl infectivity plotted as a ratio with gp120 (left) or p24 (right) concentrations. TZM-bl/gp120 ratios (shown in B, left lanel) indicate that non-mac-tropic Envs from 4 of 6 individuals are less functional compared to mac-tropic Envs (i.e. they need more Env to achieve the same level of infectivity as their mac-tropic counterparts).
