# Supplementary material for: HIV-1 non-macrophage-tropic R5 envelope glycoproteins are not more tropic for entry into primary CD4+ T-cells than envelopes highly adapted for macrophages
Source: Retrovirology. 2015 Mar 14;12:25. doi: 10.1186/s12977-015-0141-0 (PMC4373511; doi:10.1186/s12977-015-0141-0)
Supplement: Additional file 1: — Additional data and figures. Figure S1. Infection of cells using a GFP reporter+ Env+ pseudovirus. (A) HeLa TZM-bl; (B) primary CD4+ T-cells; (C) CD4+ T-cells infected following MDDC capture of virions and trans-infection. Note a GFP+ T-cell adjacent to a clump of MDDCs, which also contains additional GFP+ cells that are out of the plane of focus; (D) infected MDDC; and (E) Low level infection of MDDCs is inhibited by AZT. Note: please view electronic version of panels A-D. Figure S2. Estimation of Env+ pseudovirus infectivity for CD4+ T-cells as a percent of that measured on HeLa TZM-bl. Left panel; FFU counts from infection of primary T-cells using 2-fold dilutions of Env+ pseudovirus preparations. Right panel; Estimation of infectivity as a percent of TZM-bl using FFU counts. Figure S3. Env+ pseudovirus infectivity for Jurkat/CCR5 (A), primary T-cells (B) and MDDCs (C). For each cell type, infectivity is plotted as FFU/ml of input virus with standard deviations shown (top panels), Env+ pseudovirus infectivities as percentages of that recorded on HeLa TZM-bl are also shown as labeled points in a column scatter plot (bottom panels). Symbol colour designations are the same as described in Figure 1. Figure S4. Env+ pseudovirus infectivity for CD4+ T-cells following DEAE dextran and spinoculation. See Figure S3 for more details. Figure S5. Env+ pseudovirus infectivity for CD4+ T-cells following MDDC capture and trans-infection. See Figure S3 for more details. Figure S6. (A) Gp120 and p24 concentrations in Env+ pseudovirus preparations of late stage Envs of 6 individuals. (B) HeLa TZM-bl infectivity plotted as a ratio with gp120 (left) or p24 (right) concentrations. TZM-bl/gp120 ratios (shown in B, left lanel) indicate that non-mac-tropic Envs from 4 of 6 individuals are less functional compared to mac-tropic Envs (i.e. they need more Env to achieve the same level of infectivity as their mac-tropic counterparts). [file 12977_2015_141_MOESM1_ESM.zip › 1034352199146124_add2.pptx]

## Slide 1
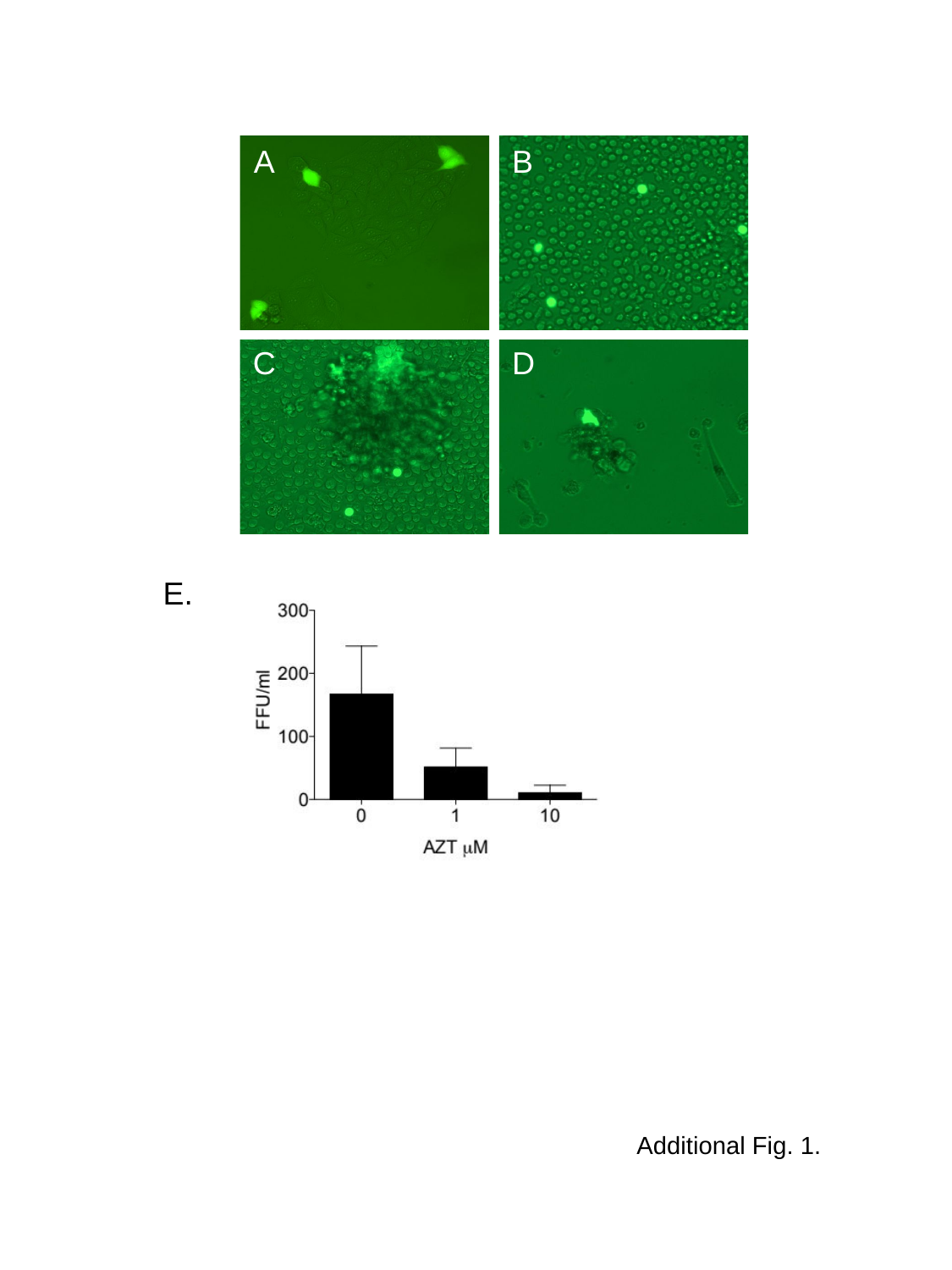

A
B
C
D
E.
Additional Fig. 1.

## Slide 2
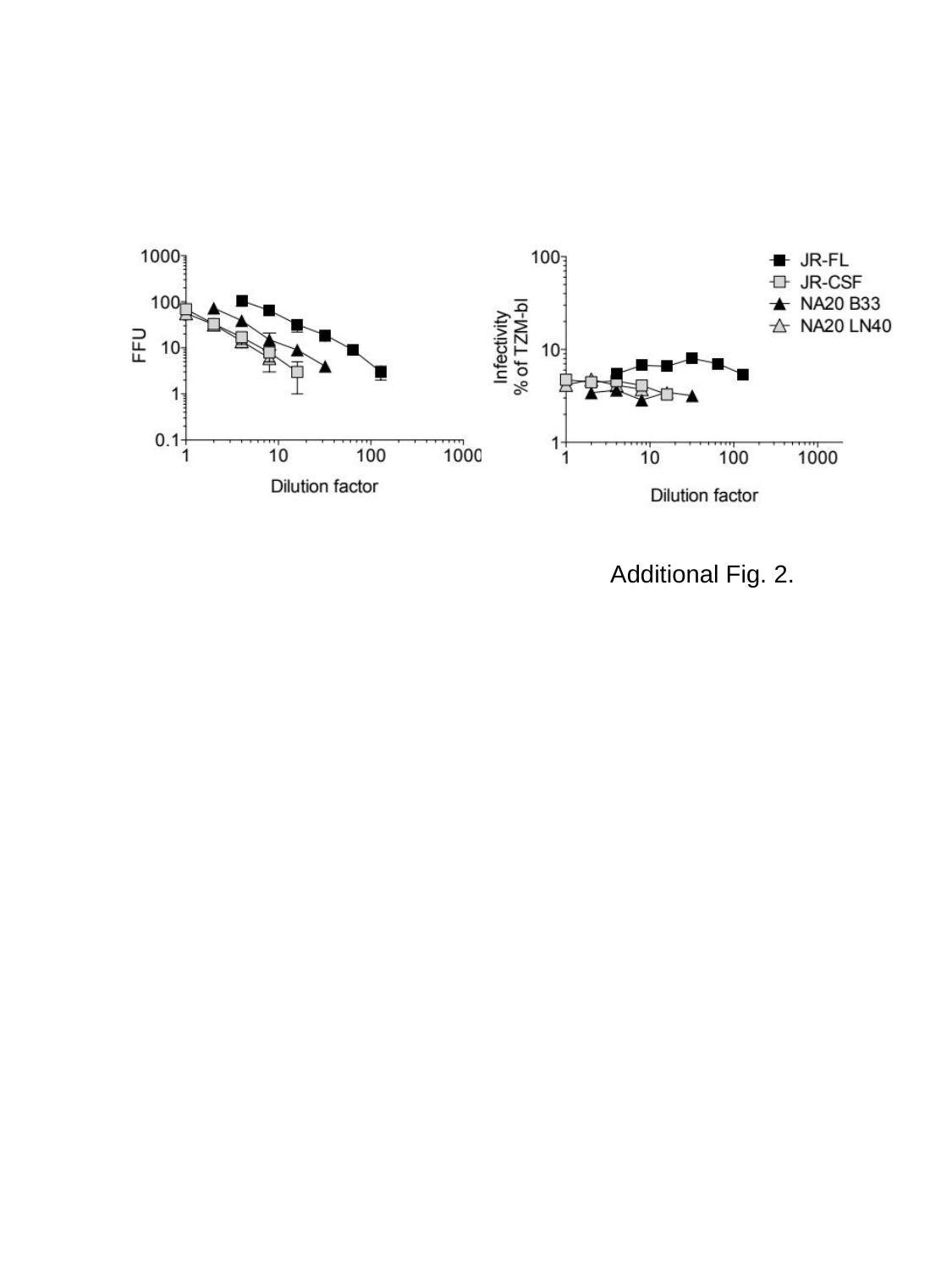

Additional Fig. 2.

## Slide 3
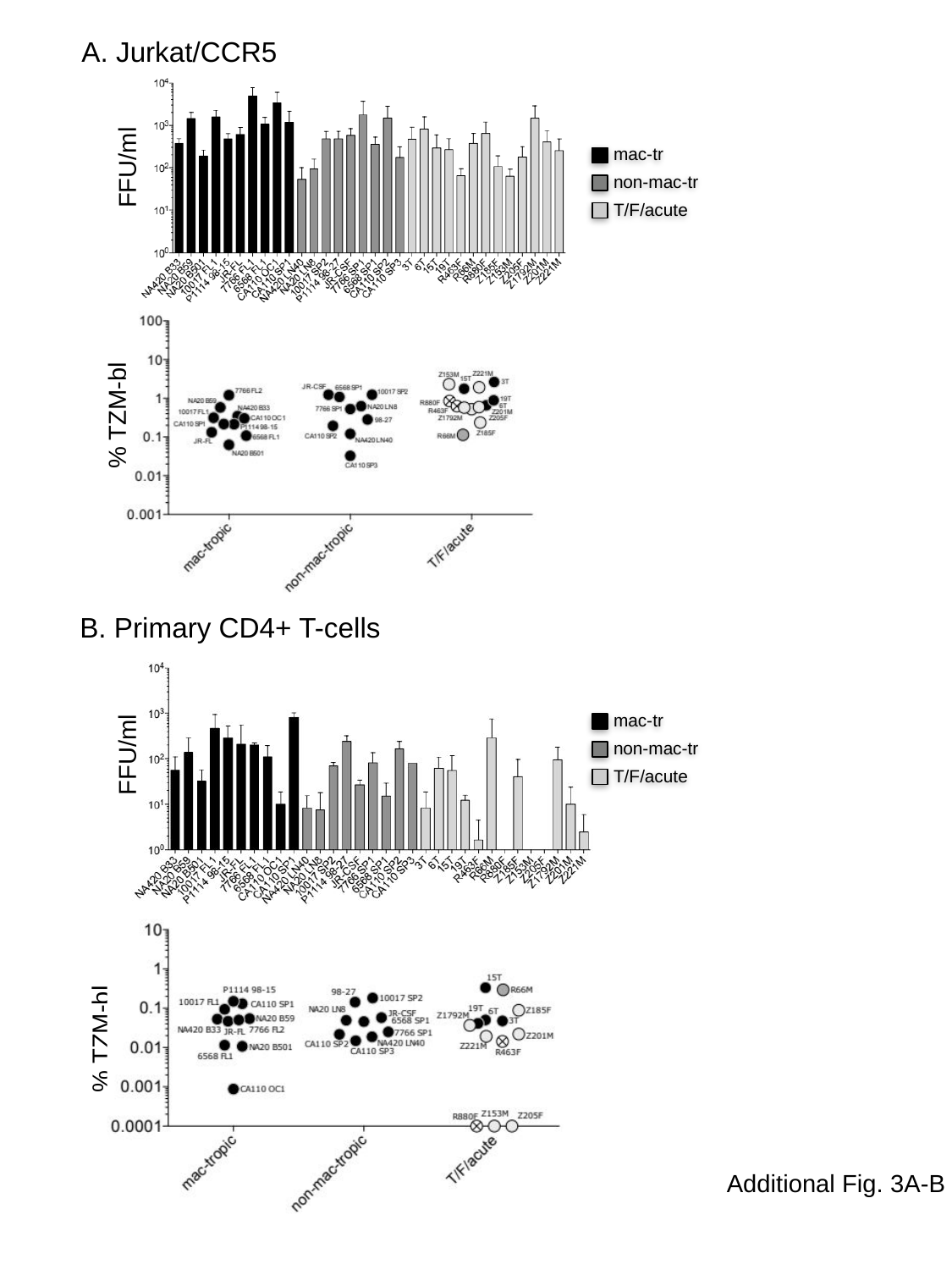

A. Jurkat/CCR5
mac-tr
FFU/ml
non-mac-tr
T/F/acute
% TZM-bl
B. Primary CD4+ T-cells
mac-tr
non-mac-tr
FFU/ml
T/F/acute
% TZM-bl
Additional Fig. 3A-B

## Slide 4
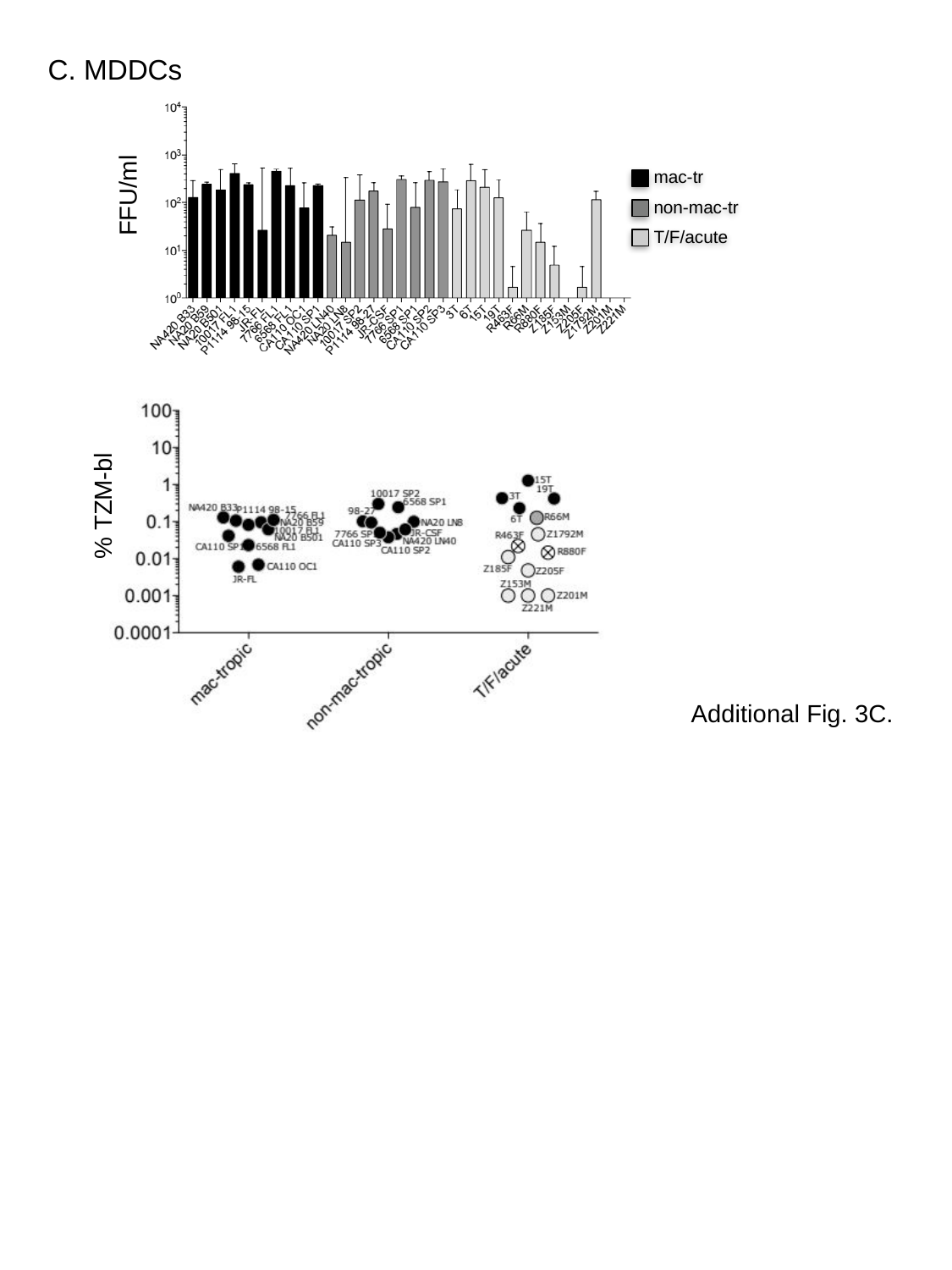

C. MDDCs
mac-tr
non-mac-tr
T/F/acute
FFU/ml
% TZM-bl
Additional Fig. 3C.

## Slide 5
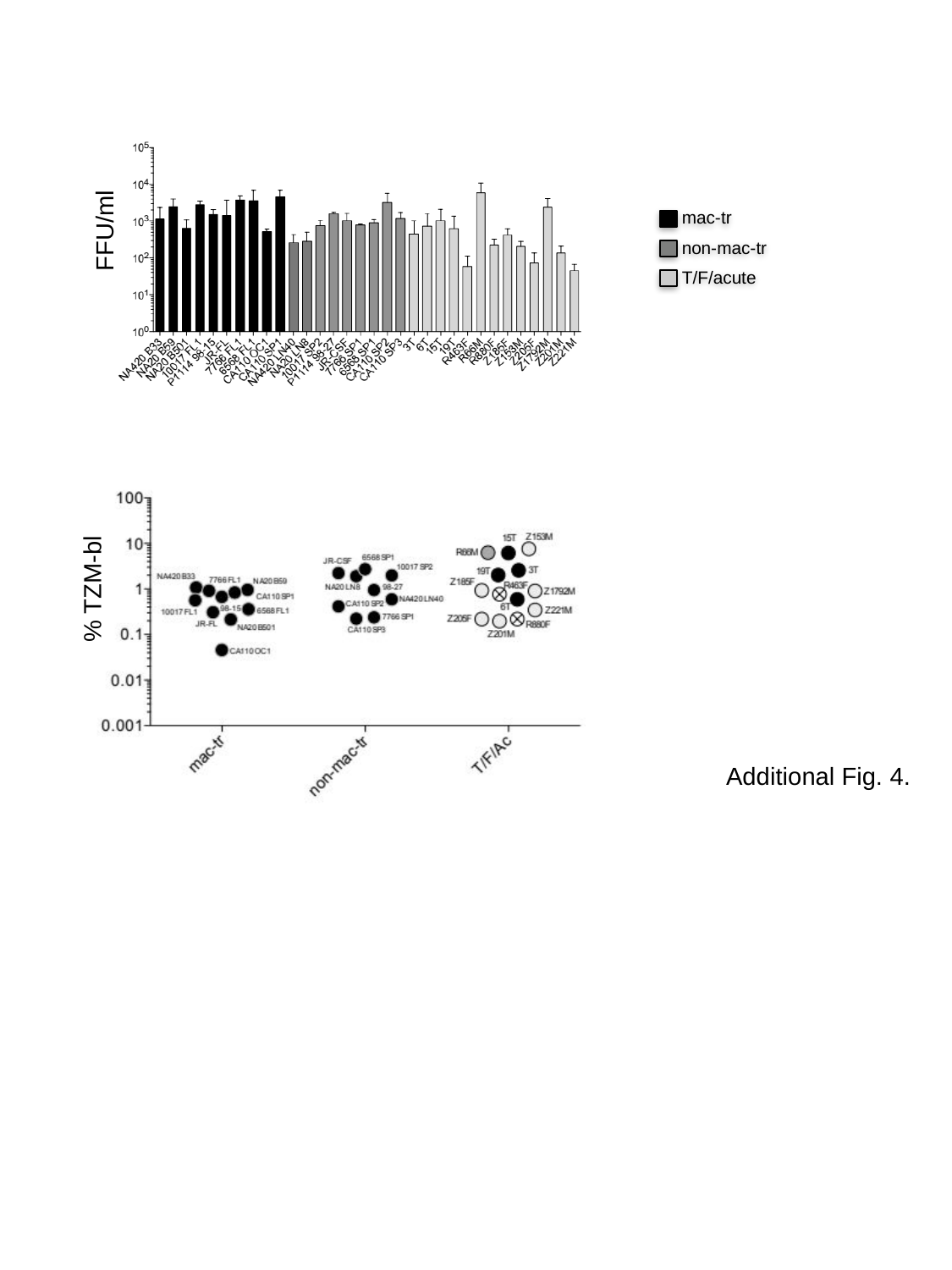

mac-tr
non-mac-tr
T/F/acute
FFU/ml
% TZM-bl
Additional Fig. 4.

## Slide 6
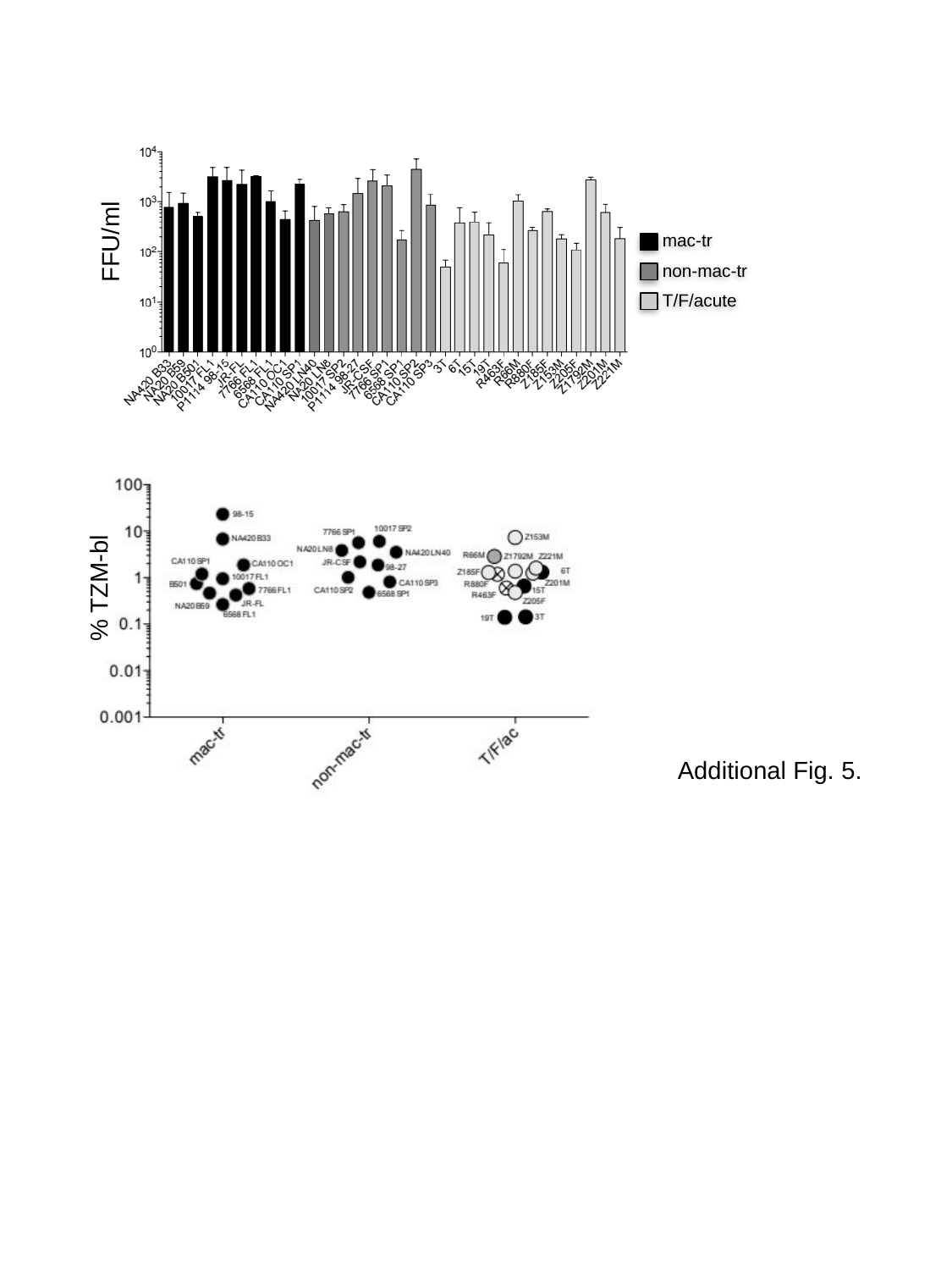

FFU/ml
mac-tr
non-mac-tr
T/F/acute
% TZM-bl
Additional Fig. 5.

## Slide 7
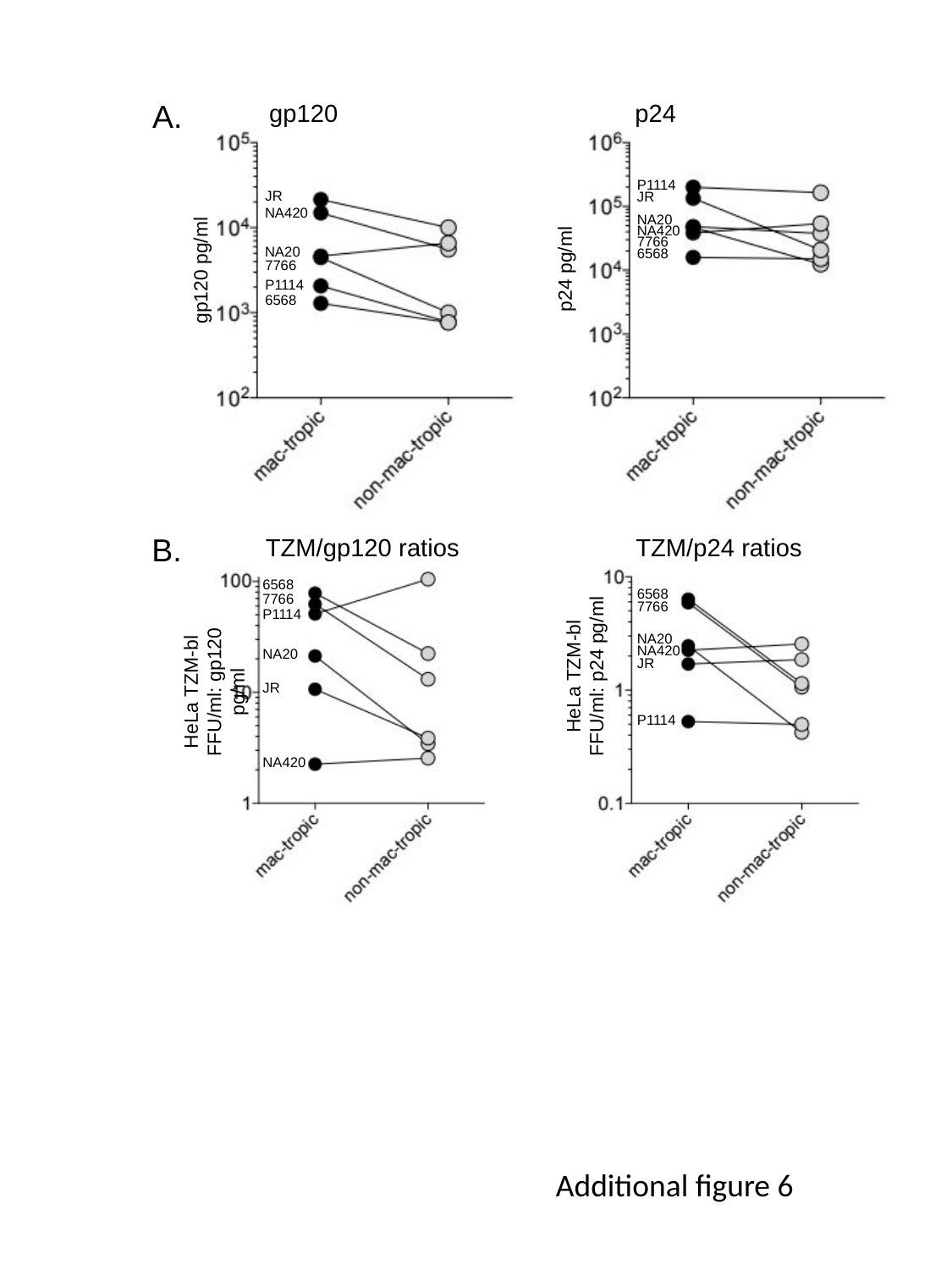

A.
p24
gp120
JR
NA420
NA20
7766
P1114
6568
P1114
JR
NA20
NA420
7766
6568
p24 pg/ml
gp120 pg/ml
B.
TZM/gp120 ratios
TZM/p24 ratios
6568
7766
P1114
NA20
JR
NA420
6568
7766
NA20
NA420
JR
P1114
HeLa TZM-bl
FFU/ml: p24 pg/ml
HeLa TZM-bl
FFU/ml: gp120 pg/ml
Additional figure 6
